# Impact of Obesity on Clinicopathologic Characteristics and Disease Prognosis in Pre- and Postmenopausal Breast Cancer Patients: A Retrospective Institutional Study

**DOI:** 10.1155/2019/3820759

**Published:** 2019-03-25

**Authors:** Nehad M. Ayoub, Rami J. Yaghan, Nour M. Abdo, Ismail I. Matalka, Laila M. Akhu-Zaheya, Alia H. Al-Mohtaseb

**Affiliations:** ^1^Department of Clinical Pharmacy, Faculty of Pharmacy, Jordan University of Science and Technology (JUST), Irbid, Jordan; ^2^Department of Surgery, Faculty of Medicine, Jordan University of Science and Technology (JUST), Irbid, Jordan; ^3^Department of Public Health and Community Medicine, Faculty of Medicine, Jordan University of Science and Technology (JUST), Irbid, Jordan; ^4^Department of Pathology and Microbiology, Faculty of Medicine, Jordan University of Science and Technology (JUST), Irbid, Jordan; ^5^Department of Adults Health Nursing, Faculty of Nursing, Jordan University of Science and Technology (JUST), Irbid, Jordan

## Abstract

**Purpose:**

To investigate the association between obesity and breast cancer clinicopathologic characteristics at presentation along with prognostic impact among Jordanian breast cancer patients. Such data are lacking in Arabian countries.

**Methods:**

In this retrospective study, 348 breast cancer patients were included. Analyses were conducted for associations between body mass index (BMI) and age at diagnosis, tumor clinicopathologic characteristics, and molecular subtypes. Eight prognostic factors were considered, and total prognostic scores were calculated. The analysis was stratified by menopausal status. Multivariate logistic stepwise regression analysis was conducted to identify predictors for breast cancer recurrence and death.

**Results:**

Mean age at diagnosis was 50.98 ± 10.96 years. Mean BMI at diagnosis was 29.52 ± 5.32 kg/m^2^. Mean age at diagnosis was significantly higher for overweight and obese patients compared to underweight/normal patients (*P* < 0.001). A significant positive correlation was observed between patient age and BMI at diagnosis (*r* = 0.251, *P* < 0.001). Grade of carcinoma was significantly correlated with BMI in the whole population examined (*P*=0.003). Obese breast cancer patients had significantly higher prognostic scores compared to nonobese cases, indicating worse prognostic features at presentation (*P*=0.034). Stratification of data analysis based on menopausal status revealed significant associations between obesity and each of tumor stage and grade among postmenopausal but not premenopausal patients (*P*=0.019 and *P*=0.031, respectively). Similarly, postmenopausal obese patients had significantly higher prognostic scores compared to nonobese counterparts (*P*=0.007), indicating worse prognosis, a finding which was also absent among premenopausal breast cancer patients. No significant association between BMI with expression status of hormone receptors, HER2, lymphovascular invasion, and molecular subtypes was found among patients. BMI was a significant predictor for disease recurrence in which obese breast cancer patients had greater odds (2-fold) to develop locoregional and distant recurrence compared to nonobese cases (*P*=0.011).

**Conclusions:**

Obesity was associated with advanced stage and grade of breast carcinoma at diagnosis. The impact of BMI on clinicopathologic characteristics and prognosis was confined to postmenopausal cases. Jordanian obese breast cancer patients are at greater risk of breast cancer recurrence and reduced survival compared to their nonobese counterparts.

## 1. Introduction

Worldwide, breast cancer is the most commonly diagnosed carcinoma among women [1, 2]. In 2012, 1.76 million breast cancer cases were diagnosed globally, accounting for a quarter of all newly diagnosed cancer cases [3]. In keeping with this, breast cancer is the leading tumor type among Jordanian women, accounting for 37.3% of all newly diagnosed female cancers [4, 5]. Several modifiable lifestyle factors can influence the risk of development and the outcome of patients with breast cancer [6]. However, the role of obesity in relation to breast cancer has captured much attention and become an increasingly growing area of research in the scientific literature.

Obesity is characterized by excess accumulation of adipose tissue [7–9]. According to the World Health Organization (WHO), obesity is defined as having a body mass index (BMI) of 30 kg/m^2^ or more [10]. At present, obesity is an established risk factor for epithelial cancers [11]. Recent epidemiological and clinical evidence confirmed that obesity is associated with increased breast cancer risk and resistance to breast cancer treatment [12–14]. However, most findings have been demonstrated in particular populations, such as patients with locally advanced disease and postmenopausal cases [15]. Although it is fairly well established that obesity is an important risk factor for breast cancer among postmenopausal women [16–19], the relationship between premenopausal obesity and breast cancer remains inconclusive [20, 21], calling for additional epidemiological studies in this regard. In addition, limited number of studies assessed the association between BMI and breast cancer subtypes [22–24]. In line with this, the impact of obesity on breast cancer survival is less established, warranting further investigations [25].

The goal of this study was to analyze the association between obesity, as determined by BMI, and age at diagnosis, tumor grade, tumor stage, receptor status, lymphovascular invasion (LVI), and tumor subtypes. In addition, the prognostic impact of obesity at diagnosis was investigated, and the effect of obesity on disease recurrence and death in breast cancer patients was further analyzed. The analysis was stratified by menopausal status. At present, there are no data available on the relationship between obesity and breast cancer in the Arab population. To the best of our knowledge, this is the first study to explore the above described associations among Jordanian women as a model for Arabian women suffering from breast cancer.

## 2. Methods

This retrospective study was conducted at Jordan University of Science and Technology (JUST) and its affiliated King Abdullah Teaching University Hospital (KAUH). KAUH is the tertiary referring center serving the four major governorates in the North of Jordan. It includes a dedicated breast unit providing comprehensive breast care facilities including early detection, various treatment modalities, and research activities. The study was approved by the Institutional Review Board (IRB) of JUST and KAUH (Research number 33/80/2014).

### 2.1. Data Collection

Review of the medical records and the electronic database, over an eleven year period from 2004 to 2014 inclusive, revealed 448 patients with the diagnosis of histologically proven breast carcinoma. However, one hundred patients who were missing more than 20% of variables of interest (22%) were excluded from final analysis resulting in a final sample size of 348 breast cancer patients who were included in this study. Patients included in the study were women diagnosed with primary breast tumors, which were confirmed histologically by Pathology Department in KAUH. Breast cancer patients who were referred from other hospitals/institutions were not included in this analysis. In addition, patients whose first presentation indicated recurrent breast tumors were excluded from this study. Collected data included demographic information, lifestyle factors, reproductive history, anthropometric variables, and tumor pathological data. Menopausal status for the patients was extracted from medical records and recorded as indicated. Menopause is defined as the permanent cessation of menstruation due to loss of ovarian follicular activity [26]. Retrospectively, menopause is defined as the time of the final menstrual period followed by at least twelve months of amenorrhea. Postmenopause describes the period following menopause regardless of whether the menopause was induced or spontaneous [26–28].

BMI was calculated using the standard method as weight in kilograms divided by the square of height in meters. Patients were divided into four groups based on WHO were classification for obesity [10]. WHO defines BMI classes as underweight (BMI < 18.5 kg/m^2^), normal (BMI 18.5–24.99 kg/m^2^), overweight (BMI ≥ 25.00 kg/m^2^), and obese (BMI ≥ 30.00 kg/m^2^) [10]. A small number of patients (*n* = 7) were classified as underweight; therefore, the “underweight” and “normal” BMI classes were categorized together as one unit for the purposes of the current analysis [19].

Tumor characteristics for patients were extracted from relevant pathology reports issued by Pathology Department at KAUH at time of diagnosis. Reports included details of tumor grade and stage, ipsilateral axillary lymph node status, LVI, detailed histological criteria, and tumor subtypes. Expression status of estrogen receptor (ER) and progesterone receptor (PR) was determined using immunohistochemical methods. Activity greater than 1% was considered positive for each hormone receptor. Immunohistochemical scores of 0 or +1 were considered negative while a score of +3 was positive for human epidermal growth factor receptor 2 (HER2/*neu)* over expression. For equivocal results of +2 score, fluorescence in situ hybridization (FISH) analysis positive for gene amplification was considered to be positive for HER2 expression. Stage of disease was classified according to the tumor-node-metastasis (TNM) cancer staging systems of the American Joint Committee on Cancer (AJCC) [29]. Grade of carcinoma was determined based on the Nottingham Combined Histologic Grade system [30]. Accordingly, tumors were classified into grade I (well-differentiated/low grade), grade II (moderately differentiated/intermediate grade), and grade III (poorly differentiated/high grade) carcinomas. Breast cancer was further classified into subtypes based on the expression of hormone receptors and HER2 status [31–33] including luminal A (ER+ and/or PR+, HER2−); luminal B (ER+ and/or PR+, HER2+); HER2-positive (ER−, PR−, HER2+); and triple-negative (ER−, PR−, HER2−).

### 2.2. Prognostic Factors and Scores

In order to investigate the potential impact of BMI on disease prognosis, the patient cohort was divided into two groups based on BMI; nonobese (BMI < 30) and obese (BMI ≥ 30). A prognostic score was calculated by considering patient and tumor characteristics at time of diagnosis. Eight prognostic factors were considered including age, tumor size, tumor grade, lymph node status, LVI, ER, PR, and HER2 status [34]. For each of these factors, the patient was assigned a score value which was eventually combined to generate a total prognostic score. Scoring details are shown in Table 1. Higher prognostic scores predict worse prognosis for breast cancer patients at disease presentation.

### 2.3. Statistical Analysis

Data analysis was performed using IBM SPSS statistical package (IBM Corp. Version 21.0. Armonk, NY, USA). Continuous variables are expressed as mean ± standard deviation or standard error of the mean. Categorical variables are presented as frequency and percentages (*n*, %). To compare categorical variables between groups, Pearson's *χ*^2^-test of independence was used. Independent sample *t*-test was applied to compare two independent groups. One-way analysis of variance (ANOVA) test was used for multiple comparisons between independent groups when indicated. To assess correlations between continuous variables, Pearson's correlation test was applied. A multivariate logistic stepwise regression was used to identify significant predictors for two disease outcomes separately, which are breast cancer recurrence and overall death. Accordingly, all variables which were included in the regression were entered into the stepwise regression with an assumed entry of 0.15 and exit significance level of 0.15. Variables which entered the regression were age, BMI, stage, grade, menopausal status, receptor status, histology, and LVI. All *P* values were two-sided at an *α* value of 0.05. Statistical differences were considered to be statistically significant at a *P* value of less than 0.05.

Dichotomization of some categorical variables was considered for correlation analysis of some study variables. This dichotomization was based on sample size and was performed in advance of conducting statistical analysis in order to avoid small sample size upon further stratification of data by menopausal status [19, 31]. Therefore, the TNM stage of breast cancer was dichotomized as early stages (I/II) and advanced stages (III/IV). Histologic grade was further categorized into grades (I/II) and grade (III) tumors. The histological type was dichotomized into invasive ductal carcinoma as one group and other types. Categories of these tumor variables were selected based on cut points previously published by other researchers [13, 15, 35].

## 3. Results

### 3.1. Overall Description of Study Population

Table 2 lists the demographic and clinical characteristics of breast cancer patients. Mean age at diagnosis was 50.98 ± 10.96 years (ranging from 24 to 83 years). One hundred and fifty-three patients were premenopausal (44%) and 195 were postmenopausal (56%). Of the 348 patients identified, 64 were classified as normal or underweight (18.4%), 118 were overweight (33.9%), and 166 were obese (47.7%). The average value of BMI at diagnosis was 29.52 ± 5.32 kg/m^2^ (range 15.9–45.5). Family history of breast cancer was negative in 278 (79.9%) patients. Forty-six percent of the study participants had advanced disease at diagnosis (stages III and IV). The majority of patients (52.3%) presented with high-grade tumors (grade III). LVI was identified in 256 (73.6%) patients. Majority of patients (91.4%) had modified radical mastectomy, while a smaller proportion (5.2%) had breast-conservation surgery. Luminal A was diagnosed in 213 (61.2%) patients. Chemotherapy was administered to 81.0% of the patients. Further description of histologic type, receptor status, tumor size, and lymph node involvement is shown in Table 2.

### 3.2. Association between BMI and Clinicopathologic Characteristics of Breast Cancer Patients

Tumor characteristics of study population stratified by BMI category are shown in Table 3. Patients were divided into three groups based on their BMI. One-way ANOVA analysis showed a significant difference in mean age at presentation between the different BMI groups (*P* < 0.001). Mean age at diagnosis was significantly higher for overweight and obese patients compared to underweight/normal patients (*F* = 19.484, *P* < 0.001). Pearson's correlation analysis revealed a significant positive correlation between patient age and BMI at diagnosis (Pearson's coefficient, *r* = 0.251, *P* < 0.001). There was a significant association between BMI and menopausal status (*P* < 0.001). Stage of disease at presentation was not significantly associated with BMI among the three groups (*P*=0.248). Tumor grade was significantly associated with BMI at presentation (*P*=0.003). No statistically significant associations were found between tumor histology or LVI and BMI (Table 3).

### 3.3. Association between BMI and Clinicopathologic Characteristics of Breast Cancer Patients according to Menopausal Status

Association between BMI category and tumor characteristics was further evaluated in premenopausal and postmenopausal groups as shown in Table 4. Mean age at diagnosis of breast cancer among premenopausal cases was 42.05 ± 5.63 years (range 24–53). Mean BMI among premenopausal patients was 28.28 ± 5.09 kg/m^2^ ranging from 17.10 to 42.57. Among these women, BMI lacked significant association with all clinicopathologic characteristics examined (Table 4).

Mean age at presentation for postmenopausal patients was 57.98 ± 8.84 years (range 39–83). Mean BMI among postmenopausal patients was 30.49 ± 5.30 kg/m^2^ ranging from 15.90 to 45.50. Among postmenopausal cases, BMI was significantly associated with patient age at presentation (*P*=0.011). A significant association was also observed between the BMI group and stage of carcinoma (*P*=0.019). Similarly, histologic grade was significantly associated with the BMI group among these patients (*P*=0.031). Independent sample *t*-test analysis comparing BMI values between premenopausal and postmenopausal groups showed postmenopausal cases to have significantly higher values of BMI at presentation compared to premenopausal cases (*t* = −3.961, *P* < 0.001). Further associations between BMI category and tumor characteristics based on menopausal status are shown in Table 4.

### 3.4. Association between BMI with Receptor Status and Molecular Subtypes in Breast Cancer Patients

**Figure 1:**
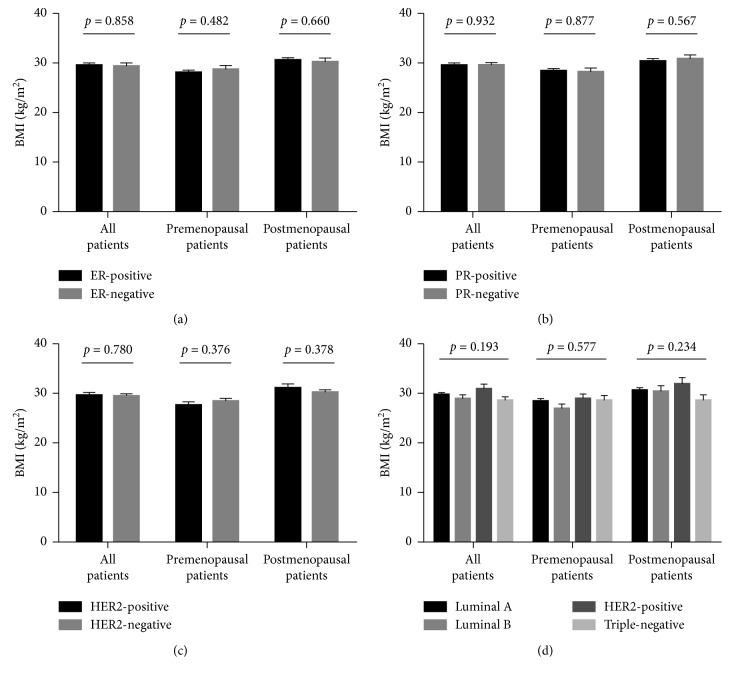
Association of BMI with receptor status and molecular subtype in breast cancer patients at time of diagnosis. BMI among all patients, premenopausal cases, and postmenopausal cases in relation to (a) ER, (b) PR, (c) HER2, and (d) molecular subtype. Bars represent mean BMI value ± SEM. BMI, body mass index; ER, estrogen receptor; HER2, human epidermal growth factor receptor 2; PR, progesterone receptor.

The association between BMI and each of hormone and HER2 receptors are shown in Figure 1. Independent sample *t*-test revealed no statistically significant difference in mean BMI values among receptor-positive and receptor-negative breast cancer patients for ER, PR, and HER2 (Figures 1(a)–1(c)). The same pattern was found when receptor expression status was further stratified based on the menopausal status (Figures 1(a)–1(c)). One-way ANOVA revealed no significant differences of mean BMI values between the different molecular subtypes of breast cancer (*F* = 1.585, *P*=0.193, Figure 1(d)). Similar findings were observed among pre- and postmenopausal patients. Chi-square analysis for associations between the receptor status and molecular subtypes with BMI category also revealed no statistically significant associations (data not shown).

### 3.5. Impact of BMI on Prognostic Features of Breast Cancer Patients

**Figure 2:**
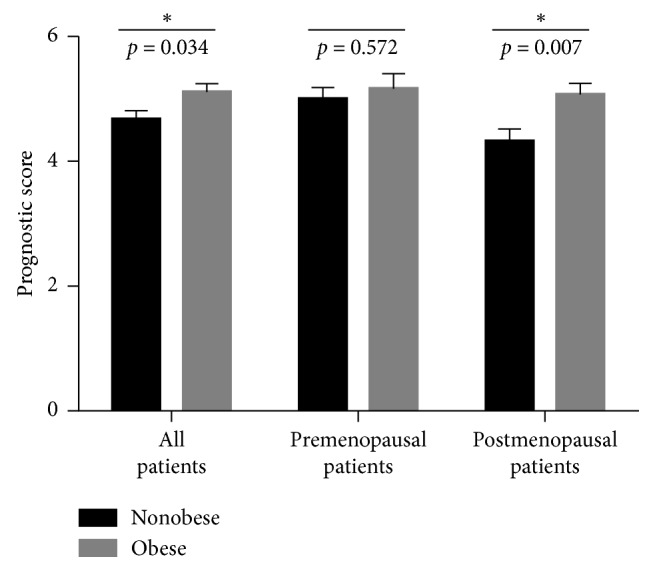
Association of obesity with prognostic scores in breast cancer patients at time of diagnosis. Bars represent mean prognostic score value ± SEM. ^*∗*^Statistical significance at *P* < 0.05.

Eight different prognostic factors were considered to generate a total prognostic score ranging from 0 to 10 in which greater scores indicated worse prognosis for breast cancer patients at time of diagnosis. In this regard, 182 (52.3%) patients were classified as nonobese, while 166 (47.7%) patients were obese. The average prognostic score was 4.89 ± 1.88 (range 0–9).  Figure 2 represents prognostic scores among obese and nonobese breast cancer patients at time of diagnosis. Independent sample *t*-test analysis indicates that obese patients have significantly higher prognostic scores compared to nonobese cases (*t* = −2.134, *P*=0.034). When prognostic scores were further compared based on menopausal status, postmenopausal obese patients were found to have significantly greater prognostic scores compared to nonobese counterparts (*t* = −2.742, *P*=0.007). Nevertheless, no statistical difference was observed for scores among obese and nonobese premenopausal patients (Figure 2).

### 3.6. Effect of Obesity on Disease Recurrence and Death in Breast Cancer Patients

The impact of obesity at diagnosis on disease recurrence and death among breast cancer patients was analyzed using multivariate logistic stepwise regression and is shown in Table 5. Based on hospital records, 16 patients (4.6%) had died after initial diagnosis of breast cancer out of 348 patients included in this study at the time of data collection. Recurrent disease was reported in 56 patients (16%) including both locoregional and distant recurrence. The stepwise regression method for recurrence found that BMI, grade, and LVI were significantly predictive of breast cancer recurrence. The odds of disease recurrence are two-fold greater among breast cancer patients who are obese compared to their nonobese counterparts (*P*=0.011). Similarly, patients with greater grade of breast carcinoma have greater odds of recurrence compared to patients with low grade (*P*=0.0115). The presence of LVI is also a significant predictor of disease recurrence (Table 5). Stage and BMI were found by multivariate logistic regression as the only predictors for death in breast cancer patients. Advanced stage at diagnosis is a significant risk factor predictive for death among patients in multivariate regression (Table 5).

## 4. Discussion

Obesity is a global health problem. In 2016, more than 1.9 billion adults were overweight, and of these, over 650 million were obese [36]. In Jordan, a recent report revealed an overall prevalence of overweight of 30% [37]. Obesity was found in 38.8% of Jordanian women aged between 15 and 49 years [37]. These local findings are in concordance with recent epidemiologic studies which indicated an alarming epidemic of obesity among women in most of the Eastern Mediterranean Region (EMR) countries, especially the Gulf Cooperation Council (GCC) countries [38]. In parallel, breast cancer has been constantly reported as the most common malignancy among Arab women in EMR [38]. Earlier studies indicated that obesity is a risk factor for breast cancer in Arab women [38, 39]. However, limited data are available regarding the impact of obesity on pathologic features of breast cancer and/or outcomes among Arab women. Obesity may be associated with increased risk of development and progression of breast cancer through several overlapping pathways. Molecular mechanisms for obesity-mediated tumorigenesis include increased aromatase-mediated estrogen production, high-circulating insulin/insulin-like growth factor I (IGF-I), and altered adipokine concentrations and their signaling pathways which collectively promote a chronic inflammatory condition [40, 41].

The link between obesity and breast cancer is complex and has been largely investigated in the literature. In our study, a positive family history of breast cancer was reported by 20.1% of patients, suggesting a potential impact of life style factors. In the Jordanian community, alcohol consumption and tobacco smoking are not common practices among women because of religious and cultural considerations. In this study, the vast majority of breast cancer patients (81.6%) were overweight or obese at diagnosis. Though many studies had previously evaluated prevalence of overweight and obesity among breast cancer patients at presentation [8, 19, 42, 43]; the percentage of overweight/obese cases among this sample of Jordanian patients was relatively greater. Patients who had higher BMI in our analysis tended to be older at diagnosis, and thus more likely to be postmenopausal. These results were consistent with earlier findings by other research groups who indicated a greater proportion of obese women among older breast cancer patients [8, 22, 44].

Grade of breast carcinoma was significantly associated with the BMI group in our study. This finding is in agreement with multiple earlier studies, suggesting an association between obesity and more aggressive breast cancer phenotypes [19, 45, 46]. Advanced presentation of obese breast cancer patients could be related to low rates of breast cancer screening among obese women [47, 48] and the possibility that obese patients have more biologically aggressive tumors as indicated by higher expression of cellular proliferation markers [46]. Expression status of ER, PR, and HER2 did not differ by the BMI group in our population. In agreement with this finding, Gillespie et al. and Kamineni et al. showed that obesity was not associated with hormone receptor or HER2 status [46, 49]. However, BMI was associated with hormone receptors in other reports [35, 42]. Data from this study did not reveal a significant association between LVI and obesity. Though these results are consistent with findings by Haakinson et al., other groups demonstrated higher rates of angiolymphatic invasion among obese patients with breast cancer at presentation [43, 49].

Strong evidence from epidemiological studies supports the fact that obesity is an established risk factor for breast cancer among postmenopausal women [50, 51]. Nevertheless, associations between BMI and premenopausal breast cancer were less consistent. A modest majority of studies reported that premenopausal obesity is inversely related to breast cancer risk and suggested a protective effect [52]. However, few studies reported either no association or a positive association between obesity and breast cancer among premenopausal women [17]. Findings in this study showed that BMI was associated with advanced stage and grade of breast carcinoma among postmenopausal but not premenopausal cases. These findings further support existing evidence of greater impact of obesity in postmenopausal breast cancer patients [16–19]. In consistence with previous studies [8, 22], our data showed a lack of association between obesity and breast cancer molecular subtypes, regardless of menopausal status.

The association between breast cancer prognosis and obesity is not as strong as with obesity and risk of breast cancer. In this regard, our study provides new insights into the impact of obesity on prognosis and disease outcomes among Arab breast cancer patients. Results revealed worse prognostic impact among obese cases as indicated by greater prognostic scores compared to nonobese patients. Interestingly, this impact was remarkably observed among obese postmenopausal but not premenopausal women compared to their nonobese counterparts. Factors included in our analysis are well correlated to prognosis in breast cancer patients and are of clinical relevance as prognostic and/or predictive factors [34, 53]. Furthermore, our data revealed that BMI is a significant predictor of disease recurrence in which obese breast cancer patients have greater odds for locoregional and distant metastases compared to nonobese cases. BMI was also a predictor of breast cancer survival in our study, but it did not reach statistical significance. This could be explained, in part, due to the small number of patients who were reported to be dead at the time of data collection. In this regard, expanding the sample size may provide better understanding of the effect of BMI on survival of breast cancer patients. The impact of obesity on breast cancer survival has been investigated among different patient populations and ethnicities. In the United States, pre- and postdiagnostic obesity, as well as postdiagnostic weight gain, were associated with greater breast cancer-specific and overall mortality in both pre- and postmenopausal non-Hispanic white women [40, 54]. Alternatively, no association was reported between prediagnostic obesity and breast cancer-specific mortality or all-cause mortality among African American women [40]. Similarly, BMI was not associated with breast cancer survival among Asian American women [55]. Analysis of associations of adult BMI with cancer mortality in geographic populations from Asia, Australia, and New Zealand within the Asia Pacific Cohort Studies Collaboration revealed a positive association between BMI and all cancer mortality among breast cancer women who were above 60 years of age [56]. Obesity has been also reported as an independent prognostic factor for distant metastases and death as a result of breast cancer among Danish women [57]. In this analysis, BMI at diagnosis was positively associated with locoregional recurrences and distant metastases as well as reduced effects of adjuvant therapy among obese breast cancer women [57]. Compelling evidence supports the association between obesity and increased incidence and risk of breast cancer development. On the contrary, the impact of obesity on breast cancer survival, recurrence, and response to therapy is inconsistent. Multiple factors could attribute to variability in research findings regarding impact of obesity on breast cancer survival. Race, ethnicity, and comorbid conditions are potential determinants of the effect of obesity on disease outcomes. In addition, variability in findings could be due, in part, to the settings in which effect of obesity was investigated. It is likely that the impact of obesity at diagnosis will be distinct from weight changes occurring at postdiagnosis phase of breast cancer.

The main limitation of this study is its retrospective nature, and thus some data were missing. Anthropometric measures were restricted to body weight and height at time of diagnosis of breast cancer. This allowed calculations of BMI, a marker of general obesity. However, visceral obesity as determined by waist circumference and waist-hip ratio could not be investigated in relation to presentation of breast cancer, as these measures were not routinely reported.

Further prospective studies to elucidate the impact of obesity on breast cancer in Jordan are mandatory. Findings in this report reflect data from some previous studies while disagree with others. Discrepancies between studies can be related to substantial variations in study designs, methods for measurements of anthropometrics, data collection, as well as potential differences in study populations. On the contrary, a point of strength in our study is the inclusion of patients from a single academic center with unified diagnostic and management protocols.

## 5. Conclusions

In conclusion, among our study group, obesity was associated with advanced stage and grade of breast carcinoma at diagnosis. Obese breast cancer patients tend to have poor prognostic features at disease presentation compared to nonobese. The impact of obesity on clinicopathologic characteristics and prognostic features was largely confined to postmenopausal cases. No association between obesity and receptor status or tumor molecular subtype was observed. Obesity is a significant predictor of disease recurrence among Jordanian breast cancer patients. Data from this report may provide insights for the impact of obesity on presentation, prognosis, and outcomes in breast cancer patients and further call for larger studies among Arab women in other countries. Findings from this study will help in developing appropriate national health plans for prevention of breast cancer and improve patient awareness of potentially modifiable lifestyle risk factors such as overweight and obesity.

## Figures and Tables

**Table 1:** Prognostic score calculations for breast cancer patients at disease presentation.

Prognostic factor	Points	Impact on overall prognosis
*Age (years)*		Patients younger than 35 years of age present with more aggressive forms of breast cancer and a worse prognosis
≥35	0	
<35	1	

*Tumor size*		The larger the tumor size, the lower rate of survival
T1	0	
T2	1	
T3	2	

*Lymph nodes*		The presence of positive lymph nodes highly influences the likelihood of recurrence
Negative	0	
Positive	1	

*Tumor grade*		Higher grade tumors are associated with higher rates of distant metastasis and poorer survival
I	0	
II	1	
III	2	

*LVI*		LVI is a poor prognostic factor representing ability of the cancer to spread via hematogenous routes
Unidentified	0	
Identified	1	

*ER*		Hormone receptor positivity is associated with higher response to endocrine therapy and a longer disease-free survival
Positive	0	
Negative	1	

*PR*		Hormone receptor positivity is associated with higher response to endocrine therapy and a longer disease-free survival
Positive	0	
Negative	1	

*HER2*		HER2 overexpression is associated with increased tumor aggressiveness, increased rates of recurrence, and increased mortality rates
Negative	0	
Positive	1	

Prognostic factors are adopted from [34]. ER, estrogen receptor; HER2, human epidermal growth factor receptor 2; LVI, lymphovascular invasion; PR, progesterone receptor.

**Table 2:** Demographic and clinical characteristics of breast cancer patients (*N* = 348).

Characteristic	*n* (%)
*Age at diagnosis* ^*∗*^ *(years)*	50.98 ± 10.96 (24–83)
<45	110 (31.6)
45–60	166 (47.7)
>60	72 (20.7)

*Marital status*	
Single	35 (10.1)
Married	301 (86.5)
Divorced	6 (1.7)
Widowed	4 (1.1)
Unknown	2 (0.6)

*Smoking status*	
Current	30 (8.6)
Past	15 (4.3)
Never	297 (85.3)
Unknown	6 (1.7)

*Alcohol intake*	
Yes	1 (0.3)
No	313 (89.9)
Unknown	34 (9.8)

*HRT*	
Yes	12 (3.4)
No	278 (79.9)
Unknown	58 (16.7)

*Menopausal status*	
Premenopausal	153 (44.0)
Postmenopausal	195 (56.0)

*BMI* ^*∗*^ (kg/m^2^)	29.52 ± 5.32 (15.9–45.5)
Underweight/normal	64 (18.4)
Overweight	118 (33.9)
Obese	166 (47.7)

*Family history of breast cancer in first-degree relatives*	
Present	70 (20.1)
Absent	278 (79.9)

*Site of disease*	
Right	159 (45.7)
Left	180 (51.7)
Bilateral	9 (2.6)

*Histologic type*	
Invasive ductal carcinoma	277 (79.6)
Invasive lobular carcinoma	36 (10.3)
Other	35 (10.1)

*Tumor stage*	
I	18 (5.2)
II	170 (48.9)
III	101 (29.0)
IV	59 (17.0)

*Tumor grade*	
I (low grade)	38 (10.9)
II (intermediate grade)	128 (36.8)
III (high grade)	182 (52.3)

*Tumor size*	
T1 (<2 cm)	38 (10.9)
T2 (2–5 cm)	220 (63.2)
T3 (>5 cm)	90 (25.9)

*Lymph node status*	
Positive	249 (71.6)
Negative	99 (28.4)

*LVI*	
Identified	256 (73.6)
Not identified	92 (26.4)

*ER status*	
Positive	258 (74.1)
Negative	90 (25.9)

*PR status*	
Positive	234 (67.2)
Negative	114 (32.8)

*HER2 status*	
Positive	84 (24.1)
Negative	264 (75.9)

*Molecular subtype*	
Luminal A	213 (61.2)
Luminal B	52 (14.9)
HER2-positive	32 (9.2)
Triple-negative	51 (14.7)

*Surgery*	
None	6 (1.7)
Mastectomy	318 (91.4)
Breast conservation	18 (5.2)
Unknown	6 (1.7)

*Adjuvant chemotherapy*	
Yes	282 (81.0)
No	26 (7.5)
Unknown	40 (11.5)

^*∗*^Mean ± SD (range). BMI, body mass index; ER, estrogen receptor; HER2, human epidermal growth factor receptor 2; HRT, hormone replacement therapy; LVI, lymphovascular invasion; PR, progesterone receptor. Other histologic subtypes included were ductal carcinoma in situ (DCIS) and mixed invasive ductal and lobular carcinoma.

**Table 3:** Clinicopathologic characteristics of breast cancer patients by BMI category (*N* = 348).

Characteristic	Normal weight (*n* = 64)	Overweight (*n* = 118)	Obese (*n* = 166)	*P* value
Age at diagnosis^a^ (years)	43.73 ± 9.23	51.83 ± 11.19	53.16 ± 10.29	**<0.001** ^*∗*^
BMI^a^ (kg/m^2^)	22.25 ± 2.24	27.76 ± 1.39	33.57 ± 4.12	**<0.001** ^*∗*^

*Menopausal status*				**<0.001** ^*∗*^
Premenopausal	43 (67.2)	52 (44.1)	58 (34.9)	
Postmenopausal	21 (32.8)	66 (55.9)	108 (65.1)	

*Histologic type*				0.191
Invasive ductal carcinoma	56 (87.5)	90 (76.3)	131 (78.9)	
Other	8 (12.5)	28 (23.7)	35 (21.1)	

*Tumor stage*				0.248
Early (I/II)	38 (59.4)	68 (57.6)	82 (49.4)	
Advanced (III/IV)	26 (40.6)	50 (42.4)	84 (50.6)	

*Tumor grade*				**0.003** ^*∗*^
I and II	28 (43.8)	71 (60.2)	67 (40.4)	
III	36 (56.3)	47 (39.8)	99 (59.6)	

*LVI*				0.458
Identified	49 (76.6)	82 (69.5)	125 (75.3)	
Not identified	15 (23.4)	36 (30.5)	41 (24.7)	

^a^Mean ± SD, one-way ANOVA test. Data are presented as *n* (%), unless otherwise specified. ^*∗*^Statistical significance at *P* < 0.05. BMI, body mass index; LVI, lymphovascular invasion. Other histologic subtypes included were invasive lobular carcinoma (ILC), ductal carcinoma in situ (DCIS), and mixed invasive ductal and lobular carcinoma.

**Table 4:** Association of clinicopathologic characteristics with BMI among premenopausal and postmenopausal breast cancer patients (*N* = 348).

Characteristic		Premenopausal (*N* = 153)				Postmenopausal (*N* = 195)			
		Normal weight (*n* = 43)	Overweight (*n* = 52)	Obese (*n* = 58)	*P* value	Normal weight (*n* = 21)	Overweight (*n* = 66)	Obese (*n* = 108)	*P* value

Age (years)	<50	43 (100.0)	49 (94.2)	53 (91.4)	0.153	8 (38.1)	10 (15.2)	13 (12.0)	**0.011** ^*∗*^
	≥50	0 (0.0)	3 (5.8)	5 (8.6)		13 (61.9)	56 (84.8)	95 (88.0)	

Histologic type	Invasive ductal	39 (90.7)	40 (76.9)	49 (84.5)	0.191	17 (81.0)	50 (75.8)	82 (75.9)	0.874
	Others	4 (9.3)	12 (23.1)	9 (15.5)		4 (19.0)	16 (24.2)	26 (24.1)	

Tumor stage	Early (I/II)	26 (60.5)	23 (44.2)	32 (55.2)	0.262	12 (57.1)	45 (68.2)	50 (46.3)	**0.019** ^*∗*^
	Advanced (III/IV)	17 (39.5)	29 (55.8)	26 (44.8)		9 (42.9)	21 (31.8)	58 (53.7)	

Tumor grade	I and II	17 (39.5)	30 (57.7)	22 (37.9)	0.079	11 (52.4)	41 (62.1)	45 (41.7)	**0.031** ^*∗*^
	III	26 (60.5)	22 (42.3)	36 (62.1)		10 (47.6)	25 (37.9)	63 (58.3)	

LVI	Identified	32 (74.4)	38 (73.1)	40 (69.0)	0.811	17 (81.0)	44 (66.7)	85 (78.7)	0.164
	Not identified	11 (25.6)	14 (26.9)	18 (31.0)		4 (19.0)	22 (33.3)	23 (21.3)	

Data are presented as *n* (%).^*∗*^Statistical significance at *P* < 0.05. BMI, body mass index; LVI, lymphovascular invasion. Other histologic subtypes included were invasive lobular carcinoma (ILC), ductal carcinoma in situ (DCIS), and mixed invasive ductal and lobular carcinoma.

**Table 5:** Multivariate logistic stepwise regression analysis of factors predictive of disease recurrence and death in breast cancer patients.

Outcomes/predictors	Odds ratio	95% confidence limits		*P* value
		Lower	Upper	

*Recurrence*				
BMI	2.215	1.200	4.089	**0.0110** ^*∗*^
Grade	1.958	1.163	3.297	**0.0115** ^*∗*^
LVI	2.527	1.083	5.896	**0.0320** ^*∗*^

*Death*				
BMI	2.381	0.799	7.098	0.1102
Stage	2.447	1.328	4.747	**0.0026** ^*∗*^

^*∗*^Statistical significance at *P* < 0.05. BMI, body mass index; LVI, lymphovascular invasion.

## Data Availability

The patient data used to support the findings of this study have not been made available because of ethical considerations.
